# Bats and Viruses: Friend or Foe?

**DOI:** 10.1371/journal.ppat.1003651

**Published:** 2013-10-31

**Authors:** James W. Wynne, Lin-Fa Wang

**Affiliations:** 1 CSIRO Australian Animal Health Laboratory, Geelong, Australia; 2 Program in Emerging Infectious Diseases, Duke–National University of Singapore Graduate Medical School, Singapore; University of Florida, United States of America

Emerging infectious diseases pose a significant threat to human and animal welfare. A high proportion of emerging and reemerging infectious diseases are zoonoses derived from wildlife [1]. Bats harbour more zoonotic viruses per species than rodents and are now recognised as a significant source of zoonotic agents [2]. Henipaviruses, coronaviruses, filoviruses, and the rabies-causing lyssaviruses have all been shown to be transmissible from bats to humans—often through an intermediate host—with fatal consequences (Figure 1). Despite the obvious risk bat viruses pose to human health, it must be acknowledged that most outbreaks of bat-borne zoonotic diseases are a consequence of human activities. From an ecological perspective, bats are a remarkable and ecologically important group with many unique biological features.

**Figure 1:**
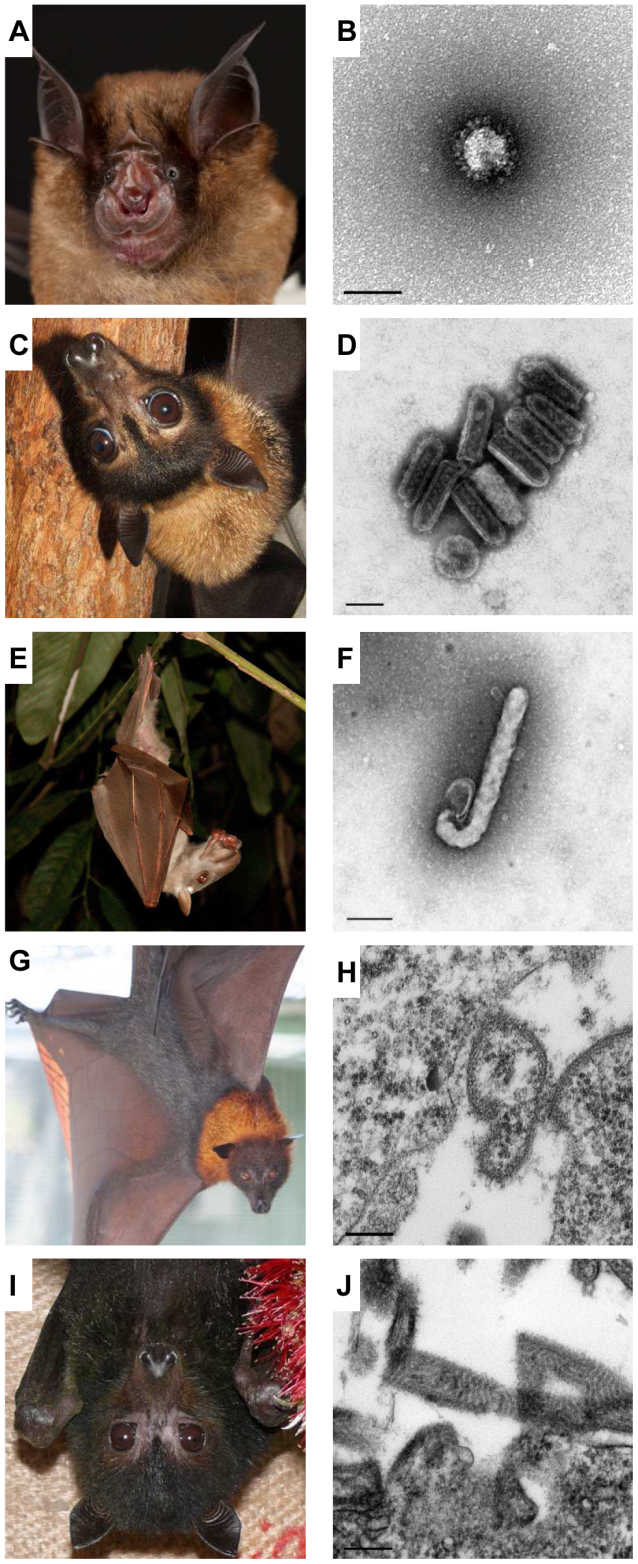
Bats are diverse, as are the viruses that infect them. The Chinese horseshoe bat (**A**; *Rhinolophus sinicus*) is one of many *Rhinolophus* sp. that are a natural host of SARS-like coronaviruses (**B**; scale bar 100 nm). The spectacled flying fox (**C**; *Pteropus conspicillatus*) along with other *Pteropus* sp. are reservoirs for the Australian Bat lyssavirus (**D**; scale bar 100 nm). A number of African fruit bats including *Hypsignathus monstrosus* (**E**) have been found to host Ebola virus (**F**; Ebola Reston, scale bar 200 nm). The Malayan flying fox (**G**; *Pteropus vampyrus*) is the natural host of Nipah virus (**H**; scale bar 200 nm). All four pteropid Australian bat species including *Pteropus alecto* (**I**) have been found to carry Hendra virus (**J**; scale bar 200 nm).

## Bats: The Only Flying Mammal

Belonging to the order Chiroptera, bats represent a remarkable example of adaptive evolution. Over 1,200 species of bats exist worldwide, making them the second most species-rich mammalian order. The Chiroptera order is classified into the suborders Yinpterochiroptera and Yangchiroptera, which represent biologically and ecologically diverse species that are distributed globally [3]. Bats have evolved an array of unique and specialised adaptations, including echolocation, hibernation, and, perhaps most extraordinarily of all, flight. Such traits have allowed specific bat species to occupy distinct ecological niches. Bats also display several unique biological features that are seemingly incompatible with their high heart rate and metabolism. These include long life span, low rate of tumorigenesis, and an ability to asymptomatically carry and disseminate highly pathogenic viruses [4].

## Bats: A Reservoir for Deadly Viruses

In many respects, bats represent the perfect reservoir for emerging zoonotic pathogens. They often live in large colonies or roosts; they can, through flight, travel and disseminate viruses over considerable distances; and they enjoy remarkable longevity for their body size. Anthropogenic activities are increasing interactions between bats, humans, and livestock, thereby heightening the zoonotic potential conferred by those characteristics. For these reasons, bats present a significant potential source of emerging infectious diseases.

The sheer number and diversity of viruses identified in bats is extraordinary and appears to be increasing almost daily. The recent identification of bat-derived viruses closely related to human pathogens, including hepaciviruses, pegiviruses [5], influenza A virus [6], hantavirus [7], and paramyxoviruses such as mumps and respiratory syncytial virus [8], are notable examples. In this review, however, we will focus on those bat viruses that have caused significant zoonotic disease outbreaks in humans and domestic animals including livestock.

### Henipaviruses

The significance of bats as a source of zoonotic disease became tragically clear with the emergence of Hendra virus (HeV) in northern Australia in 1994. In two independent spillover events, this novel paramyxovirus claimed the lives of 15 horses and of two humans who had contact with infected horses [9], [10]. Over the last few years, the incidence of HeV spillover events in Australia has drastically increased [11]. Four years after the first outbreak of HeV, another novel paramyxovirus emerged in Malaysia. Dubbed Nipah virus (NiV), this highly infectious virus was first isolated from humans and commercially farmed pigs exhibiting respiratory and neurological disease [12]. Between September 1998 and April 1999, NiV caused the death of 105 humans and the culling of over 1 million pigs in Malaysia and Singapore. NiV continues to cause regular outbreaks of encephalitis in Bangladesh and India, with evidence of direct bat-to-human and human-to-human transmission and mortality of 70–100% reported.

### Coronaviruses

Late 2002 saw one of the most high-profile examples of infectious disease emergence. The global epidemic of severe acute respiratory syndrome (SARS) ultimately caused the death of approximately 800 people. Initial efforts to identify the natural reservoir of the responsible SARS coronavirus (CoV) focused on palm civets, which had been sold in live animal markets in the Guangdong province in southern China. Subsequent research by two independent groups, however, demonstrated that civets were more likely an amplifying host and that the true reservoir of the SARS-like CoV were bats of the genus *Rhinolophus* [13], [14]. Recently, a novel CoV responsible for an acute respiratory disease (named Middle East Respiratory Syndrome, MERS) emerged [15]. To date, a total of 80 cases of human infection by the novel CoV have been reported in the Middle East, Europe, and Africa. Forty of these infections (50%) have been fatal. Genome sequencing demonstrated that this virus was most closely related to a bat CoV [16]. Furthermore, the recent identification of a highly similar MERS-like CoV from the feces of South Africa bats (Vespertilionidae family) suggests bats may also be a natural reservoir for the MERS-CoV [17], but no route of transmission from bats to humans has been identified.

### Filoviruses

Ebola and Marburg are among the most deadly viruses known to humankind. Despite their impact, the natural reservoir for these viruses has not been definitively identified. Viral RNA specific to both Ebola and Marburg has been identified in a number of fruit bat species from Gabon and Democratic Republic of Congo [18], [19]. The incidence of Marburg haemorrhagic fever in mine workers in southern Uganda, for example, was attributed to possible transmission from infected bats (*Rousettus aegyptiacus*) that had colonised the mine. Genetic analysis demonstrated that the Marburg virus isolated from the infected mine workers was highly similar to those circulating in the *R. aegyptiacus* population [20].

## Coexistence and Emergence

The strength of evidence that bats are a reservoir of zoonotic viruses is undeniable. With the exception of lyssaviruses (such as rabies), bats generally harbour viruses with no clinical signs of disease. Many species spanning the major Chiroptera suborders host zoonotic viruses, so it seems unlikely that bats' ability to asymptomatically carry viruses is a recently acquired trait. Bats and viruses have undoubtedly coevolved over millions of years. With this in mind, we would expect signatures of coevolution to be visible at the interface between bats and viruses, i.e., the innate immune system. Indeed, a number of genes involved in innate immunity were found to be under strong positive selection in the recently sequenced genomes of the Australian black flying fox (*Pterpous alecto*) and David's Myotis (*Myotis davidii*) compared to their orthologs in seven other mammalian species [21]. Accelerated evolution of innate immune genes may be a direct consequence of prolonged viral exposure, and therefore reflects the evolutionary adaptations that have led to the superior antiviral phenotype bats possess. The genetic arms race that exists between bats and viruses therefore appears to have reached equilibrium.

While bats have developed the ability to coexist with many different viruses, some of these viruses have proven to be highly lethal in other mammalian hosts. Spillover events are predominantly a result from anthropogenic activities, including habitat loss and human encroachment. The destruction of natural feeding and roosting habitats caused by urban sprawl or agricultural expansion has forced bats into urban and farming areas, heightening the chance of a negative interaction between bats, humans, and other animals. Nipah virus is a case in point. The combination of deforestation of pteropid bat habitat in Southeast Asia between 1997 and 1998 and the El Niño Southern Oscillation event triggered the encroachment of bats into pig farming/fruit growing areas in Malaysia, where NiV appears to have transmitted from bats to domestic pigs and subsequently to humans, with fatal outcomes for both [22].

## Understanding the Host: Bat Genomics and Immunology

All things considered, bats represent an important model species for studying the evolution of antiviral immunity. Knowledge obtained from studying bats could have broad significance in human medical research. Although evolutionary signatures of coexistence between bats and viruses exist, the mechanism/s by which bats asymptomatically maintain viruses remains unknown. Comparative genomics represents one strategy for identifying such mechanisms. To date, four bat genomes have been subjected to whole genome sequencing. The first bats to be sequenced were the large flying fox (*Pteropus vampyrus*) and the little brown bat (*Myotis lucifugus*) within the NIH-funded 29 Mammals Project [23]. While these genomes served as a valuable reference for many bat biologists, it was not until 2013 that a comprehensive genome comparison of two divergent bat species was published. Within this study, Zhang and coworkers [21] sequenced and compared the genomes of the Australian black flying fox (*P. alecto*) and David's Myotis (*M. davidii*). Pronounced genomic changes were observed in a number of immune genes in both species. For instance, all members of the PYHIN gene family, which play an important role in DNA sensing and formation of inflammasomes, appear to be lost in both bat species [21]. Other immune-related genomic changes that have occurred in bats include the contraction of the natural killer cell receptors [21]. The functional consequence of this apparent loss of this important immune system capability remains unclear.

The four bat genomes now available represent vital resources to the scientific community. When combined with transcriptome and proteome datasets, these approaches provide a powerful strategy for investigating host-pathogen interactions on a global scale.

## Do Viruses Benefit the Host?

The fact that bats harbour such a large number of viruses poses an important question: do these viruses provide any benefit to the host? In some instances, primary viral infections have the ability to prevent subsequent infections by homologous viruses. Continuous infection of bats with nonpathogenic antecedent adapted viruses may actually bestow a superior antiviral immune state against new invading viral pathogens. It has also been shown that persistent infection of herpesvirus can modulate the innate immune system of mice, resulting in protection against lethal infection of bacteria [24]. Endogenous retroviruses, like those recently identified in bats [25], may also promote a continuously activated antiviral state. Considering bats are extremely long lived for their body size and that they demonstrate low rates of tumorigenesis, it is possible that some bat viruses may have oncolytic behavior. Viruses that preferentially target tumor cells are well documented, including some herpes and reovirus members. It seems plausible that some of the viruses that bats harbour may have oncolytic properties that confer antitumor activity to the host. Additional research is required to address this speculation and to better understand and mitigate bat-derived zoonoses.
